# Acute Myocarditis: From Pathophysiology to Risk‐Stratified Management—A Clinical Review

**DOI:** 10.1111/eci.70247

**Published:** 2026-08-10

**Authors:** Michele Golino, Marco Giuseppe Del Buono, Michele Marchetta, Nicola Potere, Aldo Bonaventura, Alessandra Vecchiè, Danilo Malandrino, Giacomo Emmi, Jordana Kron, Antonio Abbate

**Affiliations:** ^1^ Robert M. Berne Cardiovascular Research Center, and Division of Cardiology University of Virginia Charlottesville Virginia USA; ^2^ Pauley Heart Center, Division of Cardiology Virginia Commonwealth University Richmond Virginia USA; ^3^ Division of Cardiology University of Florida College of Medicine ‐ Jacksonville Jacksonville Florida USA; ^4^ Department of Cardiovascular Medicine Fondazione Policlinico Universitario A. Gemelli IRCCS Rome Italy; ^5^ Department of Medicine Vanderbilt University Medical Center Nashville Tennessee USA; ^6^ Department of Medicine University of Ottawa at the Ottawa Hospital and the Ottawa Hospital Research Institute Ottawa Ontario Canada; ^7^ Department of Medicine and Ageing Sciences ‘G. D'annunzio’ University Chieti Italy; ^8^ Department of Internal Medicine, Medical Center S.C. Medicina Generale 1, Ospedale di Circolo and Fondazione Macchi, ASST Sette Laghi Varese Italy; ^9^ Department of Experimental and Clinical Medicine, Internal Interdisciplinary Medicine Unit University of Florence Italy, Careggi University Hospital Florence Italy; ^10^ Department of Medical, Surgery and Health Sciences University of Trieste Trieste Italy

**Keywords:** cardiac magnetic resonance, endomyocardial biopsy, immunosuppressive agents, myocarditis, prognosis

## Abstract

**Background:**

Acute myocarditis is an inflammatory disease of the myocardium with an annual incidence of 4–14 per 100,000 individuals, predominantly affecting young adults. Its clinical features are frequently nonspecific, mimicking acute coronary syndrome, which makes early recognition and management challenging. The disease results from infectious (predominantly viral) and noninfectious triggers, including autoimmune disorders, immune checkpoint inhibitors and mRNA vaccines. Pathophysiology involves a dysregulated interplay between innate immunity and adaptive immunity.

**Clinical Features and Diagnosis:**

Presentation is typically dominated by chest pain, with dyspnea and syncope reported less frequently. Cardiac magnetic resonance (CMR), with the 2018 updated Lake Louise criteria, has become the cornerstone of noninvasive diagnosis, whereas endomyocardial biopsy (EMB), in experienced centres, remains the gold standard for histological characterization and guiding immunosuppressive therapy.

**Outcomes and Management:**

Uncomplicated myocarditis usually resolves spontaneously. However, approximately 25% of patients with myocarditis have left ventricular systolic dysfunction, ventricular arrhythmias or acute heart failure. Mortality ranges from 1% to 7%, depending on presentation, aetiology and specific populations. Treatment centers on guideline‐directed heart failure therapy, with immunosuppression reserved for complicated presentations and virus‐negative, autoimmune or histologically specific subtypes. Mechanical circulatory support is critical in fulminant cases, where mortality is high.

**Conclusions:**

This clinical review synthesizes recent guideline updates and emerging trial data, primarily from literature published in the past 10 years, to support a phenotype‐driven approach to acute myocarditis, in which management is guided by clinical severity, suspected aetiology, selective use of CMR and EMB and targeted therapy. Ongoing trials investigating corticosteroids, targeted biologics and novel therapies may further refine personalized immunomodulatory strategies.

## Graphical abstract

Stepwise approach to patients with suspected acute myocarditis, integrating the 2024 American College of Cardiology (ACC) four‐stage classification (Stages A–D) and the 2025 European Society of Cardiology (ESC) inflammatory myopericardial syndrome (IMPS) framework. Step 1 (Initial Assessment): clinical presentation, first‐line investigations (ECG, high‐sensitivity cardiac troponin [hs‐cTn], natriuretic peptides, inflammatory markers, echocardiography) and exclusion of mimics (acute coronary syndrome, takotsubo syndrome, drug/toxin‐related injury). Step 2 (Risk Stratification): classification at presentation into uncomplicated (haemodynamically stable, LVEF ≥ 50%, no sustained arrhythmias), complicated (LVEF 30%–49%, symptomatic arrhythmias, high‐grade atrioventricular block, myopericarditis) or fulminant (cardiogenic shock, LVEF < 30%, refractory arrhythmias, severe organ hypoperfusion). Step 3 (Diagnostic Workup): cardiac magnetic resonance (CMR) as first‐line non‐invasive test using the 2018 Lake Louise criteria; endomyocardial biopsy (EMB) reserved for fulminant presentations, new‐onset heart failure < 2 weeks with haemodynamic compromise, ventricular arrhythmias/high‐grade AV block, suspected specific etiologies (giant cell myocarditis, eosinophilic, sarcoidosis, immune checkpoint inhibitor–associated) or unclear diagnosis; additional testing (viral PCR, autoimmune panel, FDG‐PET, genetic testing) as indicated. Step 4 (Management Strategy): tailored to severity and aetiology—supportive care and activity restriction for uncomplicated cases; guideline‐directed medical therapy (GDMT) for heart failure, arrhythmia management and consideration of immunosuppression for complicated presentations; intensive care monitoring, inotropes/vasopressors and early mechanical circulatory support (MCS) for fulminant disease, with early immunosuppression when specific aetiology is suspected or confirmed by EMB. Step 5 (Follow‐up and Long‐term Management): early clinical reassessment at 2–4 weeks, repeat CMR at 3–6 months, exercise restriction for ≥ 3–6 months with gradual return contingent on LVEF normalization and absence of arrhythmias, arrhythmic risk stratification and consideration of implantable cardioverter‐defibrillator (ICD) or wearable cardioverter‐defibrillator (WCD) in selected patients. AF, atrial fibrillation; AV, atrioventricular; CMR, cardiac magnetic resonance; CRP, C‐reactive protein; ECG, electrocardiogram; EMB, endomyocardial biopsy; ESR, erythrocyte sedimentation rate; FDG‐PET, fluorodeoxyglucose positron emission tomography; GCM, giant cell myocarditis; GDMT, guideline‐directed medical therapy; hs‐cTn, high‐sensitivity cardiac troponin; ICD, implantable cardioverter‐defibrillator; ICI, immune checkpoint inhibitor; IMPS, inflammatory myopericardial syndrome; LGE, late gadolinium enhancement; LVEF, left ventricular ejection fraction; MCS, mechanical circulatory support; MMF, mycophenolate mofetil; NTproBNP, N‐terminal pro‐B‐type natriuretic peptide; RWMA, regional wall motion abnormality; VF, ventricular fibrillation; VT, ventricular tachycardia; WCD, wearable cardioverter‐defibrillator (Created with ChatGPT, OpenAI).

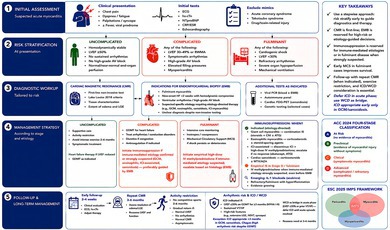

## 1 Introduction

### 1.1 Definition

Myocarditis is an inflammatory disease of the myocardium resulting in myocardial damage without an ischemic, toxic or traumatic cause [1]. It is classified by temporal course: acute (< 1 month), subacute (1–3 months) and chronic (> 3 months) [2, 3]. Inflammatory cardiomyopathy refers to myocarditis associated with cardiac dysfunction and remodeling, beyond the acute phase [4]. In some cases, the inflammation can extend to the pericardial layers in the so‐called ‘perimyocarditis’ [1]. The latest ESC guidelines have introduced the term inflammatory myopericardial syndrome, a spectrum from isolated myocarditis to isolated pericarditis through a combination of both [5].

### 1.2 Epidemiology

The annual and global incidence of acute myocarditis is 4–14 per 100,000 individuals, with a mortality rate of 1%–7% [1]. The disease predominantly affects young adults between 20 and 40 years of age, with men representing 60%–80% of cases [6]. The increased use of cardiac magnetic resonance imaging (CMR) has led to a gradual rise in reported incidence in the United States, from 9.5 to 14.4 cases per 100,000 persons [7]. Up to 95% of adult patients present with chest pain, while 19%–49% present with dyspnea and 5%–7% with syncope [1, 8, 9, 10]. About 75% of hospitalized patients have an uncomplicated course with near‐zero in‐hospital mortality.

### 1.3 Challenges and Importance of Early Detection and Management

Symptoms are nonspecific, often mimicking other cardiac conditions such as acute coronary syndrome, leading to delayed or missed diagnoses [2]. Early detection is critical, as timely intervention can prevent life‐threatening complications. CMR is essential for detecting active inflammation and monitoring disease progression over time [1, 6]. The 2024 American College of Cardiology (ACC) Expert Consensus introduced a novel four‐stage classification system (from A to D) to stratify risk and guide management, emphasizing the importance of early recognition and appropriate referral to specialized centres for high‐risk patients [2].

## 2 Pathophysiology

### 2.1 Causes: Infectious and Non‐Infectious

Viral infections represent the most common aetiology [1, 8, 9]. The spectrum of viral pathogens has shifted from predominantly adenovirus and enteroviruses (including coxsackievirus) to parvovirus B19 and human herpesvirus 6 [11]. Other viral causes include influenza, coronaviruses, cytomegalovirus, Epstein–Barr virus and human immunodeficiency virus. In selected populations, non‐viral infectious agents remain significant, including bacteria ( *Corynebacterium diphtheriae* , *Borrelia burgdorferi* ), parasites (*Trypanosoma cruzi*) and fungi [6, 12]. Non‐infectious causes include myocarditis due to autoimmune disorders [8, 13, 14], adverse reactions to medications (antibiotics, central nervous system agents, immune checkpoint inhibitors [ICIs]) and vaccines (smallpox, mRNA SARS‐CoV‐2) [1, 11].

### 2.2 Mechanisms of Inflammation: Innate and Adaptive Immunity

**FIGURE 1:**
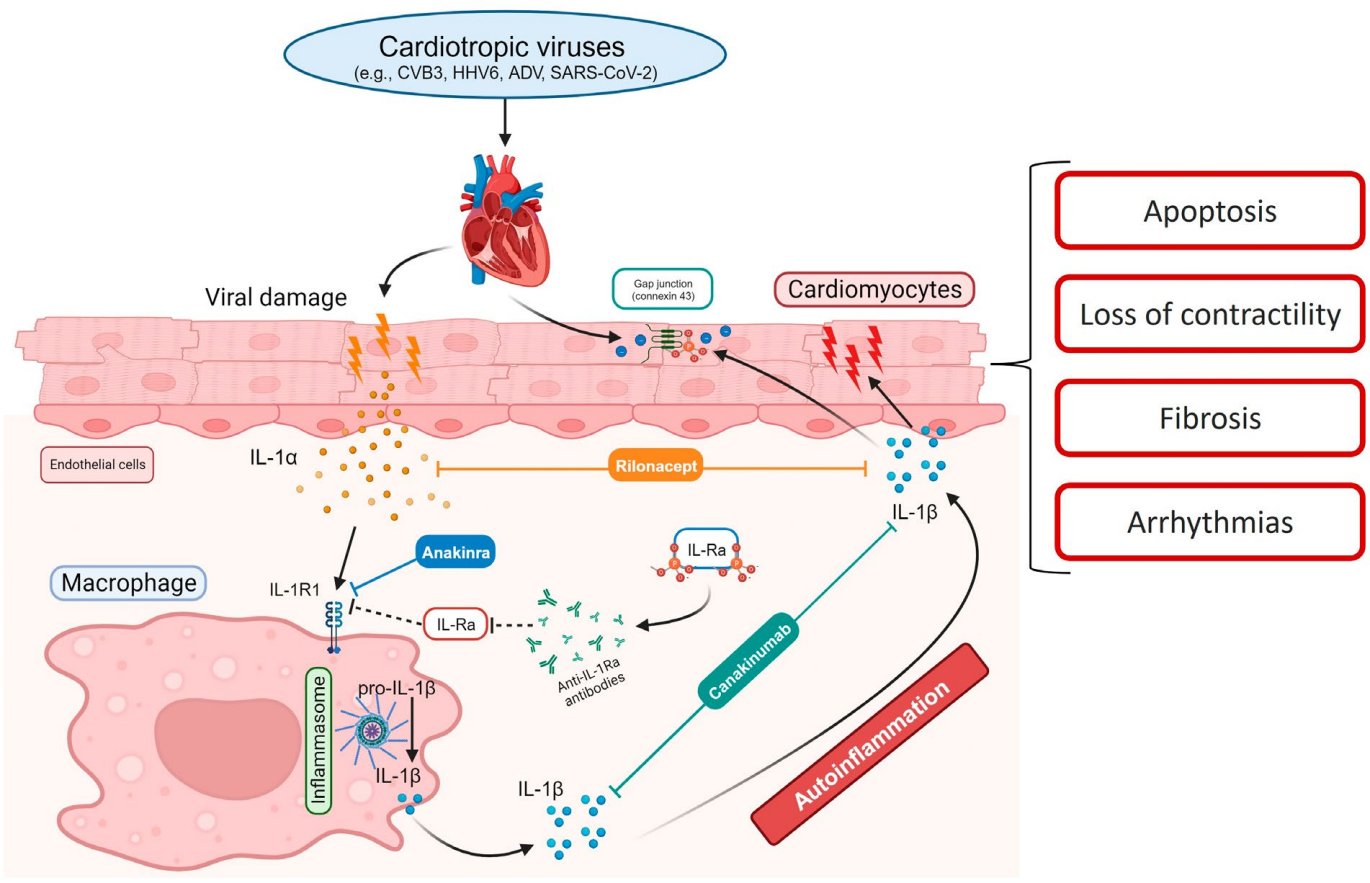
The innate immune response in acute myocarditis: The auto‐inflammatory hypothesis. Cardiotropic viruses (e.g., coxsackievirus B3 [CVB3], human herpes virus 6 [HHV6], adenovirus [ADV], severe acute respiratory syndrome‐coronavirus‐2 [SARS‐CoV‐2]) invade the myocardium via the bloodstream, causing cardiomyocyte damage. Additionally, viruses (e.g., ADV) can also lead to the phosphorylation of Connexin (Cx) 43, impairing gap junction function and increasing arrhythmic risk. Damaged cardiomyocytes release interleukin (IL)‐1α and intracellular ‘alarmins’ which in turn bind the IL‐1 receptor 1 (IL‐1R1) on macrophages, triggering the activation of a cytosolic macromolecular structure named ‘inflammasome’, responsible for the cleavage of pro‐IL‐1β into its final biologically active form IL‐1β. Once activated, IL‐1β is released into the blood and amplifies the inflammatory process by further damaging cardiomyocytes and releasing additional cytokines and ‘alarmins’ (a process referred to as ‘autoinflammation’). IL‐1β also induces Cx43 phosphorylation, further affecting direct and anisotropic electrical coupling in the heart. Over time, persistent autoinflammation can precipitate cardiomyocyte apoptosis, impaired contractility, progressive fibrosis and predispose an arrhythmogenic substrate. IL‐1 receptor antagonist (IL‐1Ra) is a natural anti‐inflammatory agent that competitively binds to IL‐1R1, blocking IL‐1 signaling. In some diseases (e.g., Multisystem Inflammatory Syndrome in Children [MIS‐C]), an atypical hyperphosphorylated isoform of IL‐1Ra induces the production of anti‐IL‐1Ra antibodies, neutralizing its anti‐inflammatory effect on the IL‐1R1 receptor. Anakinra, Rilonacept and Canakinumab are biological drugs inhibiting IL‐1 signaling. Anakinra is a human recombinant form of IL‐1Ra that directly blocks the IL‐1R1. Rilonacept is a fusion protein that combines parts of the human IL‐1R and the Fc portion of human IgG1 and acts by binding both IL‐1α and IL‐1β, effectively trapping them and preventing their interaction with cell‐surface receptors. Canakinumab is a monoclonal antibody that selectively targets and neutralizes IL‐1β (Created with BioRender.com). Reproduced from Golino et al. [15].

**FIGURE 2:**
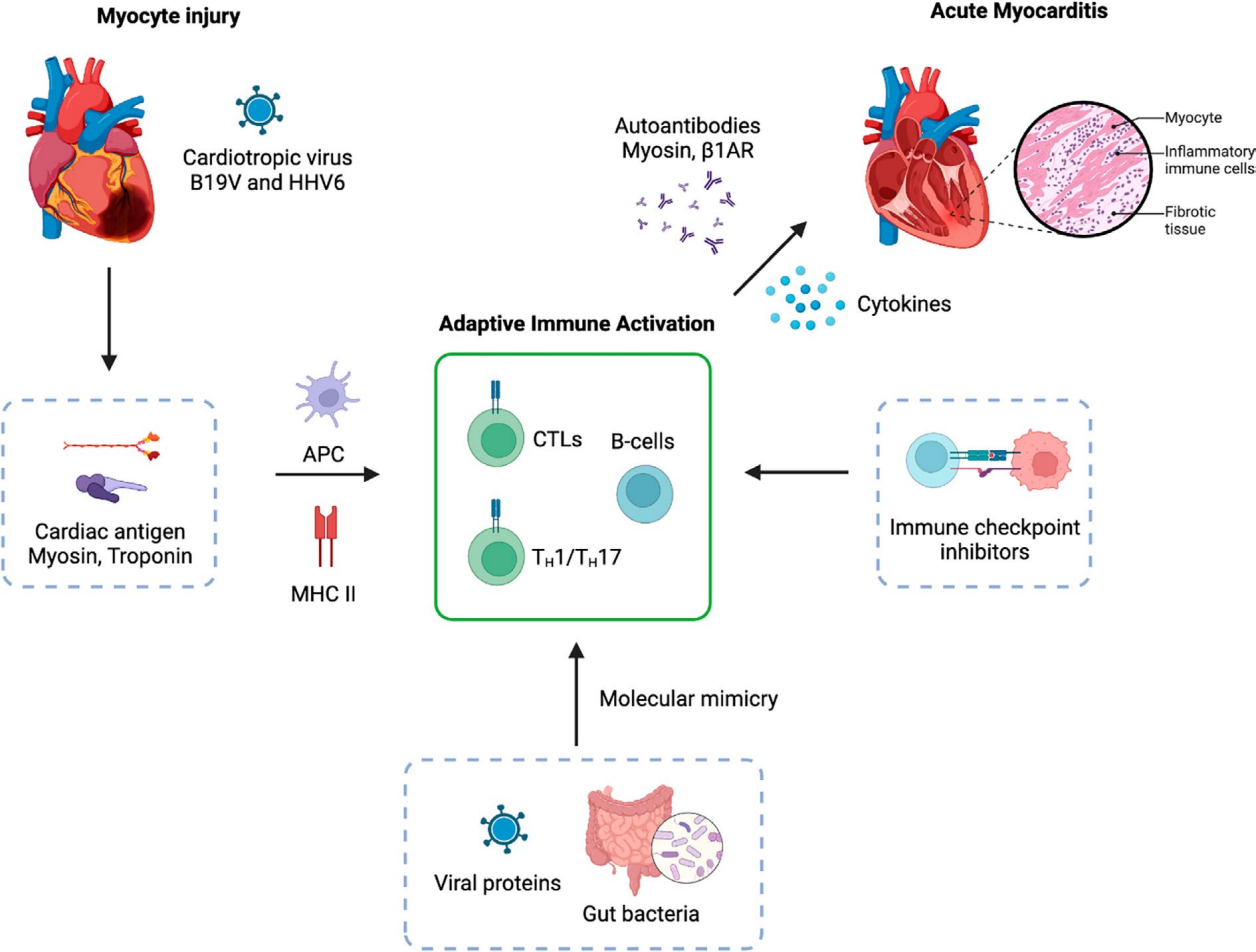
The adaptive immune response in acute myocarditis. Central to the development of acute myocarditis is the priming and activation of the adaptive immune system. This can occur via (anticlockwise from top left): Myocyte injury with subsequent release and processing of myocardial antigens, molecular mimicry of (cardiotropic) viral and gut bacterial proteins or direct activation by immune‐checkpoint inhibitors. Immune activation leads to the development of pathological B‐ and T‐cell subsets that can migrate to the heart and exacerbate inflammation by releasing cytokines, exerting direct cytotoxic effects and forming and secreting autoantibodies against myocardial proteins. B19V, parvovirus B19; HHV6, human herpesvirus 6; APC, antigen presenting cell; MHC II, major histocompatibility complex class II; CTLs, cytotoxic T‐lymphocytes; TH1/TH17, T‐helper 1/T‐helper 17 cells, β1AR, β1 adrenoreceptor (Created with BioRender.com). Reproduced from Golino et al. [15].

The innate immune system responds non‐specifically to infection or injury. Cell death activates pattern recognition receptors, including Toll‐like receptors, leading to the release of acute inflammatory mediators, including tumor necrosis factor (TNF), interleukin (IL)‐1β and IL‐6 [11, 15]. Central to this response is the NACHT, leucine‐rich repeat and pyrin domain‐containing protein 3 (NLRP3) inflammasome, which activates pro‐inflammatory cytokines like IL‐1β and IL‐18, triggering inflammation involving neutrophils and macrophages [16, 17]. In preclinical models, dysregulated inflammasome activity can lead to excessive myocardial inflammation, systolic dysfunction and tissue remodeling [16, 17] (Figure 1). The adaptive immune response evolves over hours to days as antigen‐bearing cells migrate to regional lymph nodes [18]. Antigen‐presenting cells process viral and potentially cardiac self‐antigens and present them to T‐helper cells, triggering clonal expansion of cytotoxic T cells to eliminate infected cells [19, 20]. In genetically susceptible individuals, molecular mimicry between viral and cardiac proteins, particularly α‐myosin heavy chain, may cause a breakdown of T‐cell tolerance [18, 19, 21]. This autoimmune response can persist even after viral clearance, with cardiac tissue‐resident memory T cells specific for myosin igniting disease when tolerance is breached [21]. B cells produce autoantibodies that contribute to ongoing myocardial injury [11]. IL‐1 and IL‐17A have been implicated in progression to cardiac fibrosis and dilated cardiomyopathy through activation of cardiac fibroblasts and monocyte infiltration [11, 15], providing a mechanistic rationale for emerging reports of IL‐17A inhibition with secukinumab in selected patients with psoriasis‐associated myocarditis [22] (Figure 2). Endogenous IL‐1 receptor antagonist serves as a naturally occurring brake on IL‐1‐mediated inflammation by competitively blocking IL‐1 receptor binding, though the protective mechanism may be neutralized by anti‐IL‐1RA autoantibodies, as demonstrated in myocarditis following SARS‐CoV‐2 infection and vaccination [23]. Autoimmune‐mediated injury is the most clinically actionable, as it provides the rationale for immunosuppression in specific histological subtypes (giant cell, eosinophilic, ICI‐myocarditis). Conversely, NLRP3 inhibition, broader IL‐1 blockade and IL‐17A‐targeted therapy remain investigational or limited to highly selected phenotypes.

## 3 Clinical Presentation

Acute myocarditis clinically presents with highly heterogeneous signs and symptoms, depending on the degree of organ involvement, aetiology and inter‐individual variability [1, 6, 24]. Patients are generally evaluated in the emergency room for chest pain, dyspnea, fatigue, palpitations or syncope [3, 9, 10, 25, 26]. In a large contemporary cohort of acute myocarditis, chest pain was the most frequent symptom at presentation, followed by dyspnea and syncope [8]. Pericardial involvement (i.e., perimyocarditis) occurred in up to 25% of cases [8]. Common prodromal manifestations include fever, sore throat and gastrointestinal symptoms, preceding clinically overt myocarditis by a few days or weeks [8]. Patient history should explore potential exposure to risk factors including (i) drugs (e.g., antibiotics, immune checkpoint inhibitors, clozapine); (ii) areas endemic for viruses associated with myocarditis; (iii) toxic substances (e.g., cocaine, amphetamine); (iv) infectious agents and (v) personal or family history of myocarditis and/or cardiomyopathy [27]. Most patients have an uncomplicated presentation, whereas a clinically important minority present with heart failure, ventricular arrhythmias or fulminant disease [3, 8], consistent with prior cohorts [10, 28, 29, 30]. Specific subtypes, such as giant‐cell myocarditis, more frequently present with rapidly progressive heart failure or cardiogenic shock [31]. Conduction disturbances and worsening heart failure are more frequent among subjects with sarcoidotic myocarditis and ICI‐myocarditis [32, 33]. Non‐negligible proportions of patients with acute myocarditis and complete atrioventricular block or ventricular tachycardia may have previously undiagnosed cardiac sarcoidosis [34]. The modality and severity of clinical presentation correlate with adverse clinical outcomes, being an integral part of the current risk‐based management of acute myocarditis [3, 6] (Graphical Abstract).

## 4 Diagnostic Approaches

Early evaluation should be guided by initial risk stratification and a structured diagnostic work‐up that confirms myocardial injury and inflammation, expedites advanced imaging and/or histologic assessment in higher‐risk patients and systematically excludes alternative causes. The vast majority of patients exhibit elevated troponin T, troponin I or creatine phosphokinase‐MB and up to 80%–95% have increased inflammatory biomarkers such as C‐reactive protein or erythrocyte sedimentation rate [8, 9, 35]. Neutrophil leukocytosis is common, with lymphocytosis being seen in viral or autoimmune diseases and eosinophilia suggestive of eosinophilic myocarditis [36]. Electrocardiogram abnormalities are found in about 85% of patients [8, 10]. ST‐segment elevation, especially in inferior and lateral leads, is the most frequent abnormality (56%), followed by other ST‐T segment abnormalities and bundle‐branch block [8, 37]. In younger patients without known heart disease, QRS width > 120 ms, atrioventricular block, symptomatic bradycardia or ventricular arrhythmias should raise suspicion for acute myocarditis [3].

Transthoracic echocardiography may show increased myocardial wall thickness, abnormal echogenicity, segmental hypokinesis in the inferolateral walls, diastolic dysfunction and pericardial effusion [3]. Left ventricle (LV) size is often normal initially, and LV ejection fraction is preserved in three‐quarters of patients at the time of presentation [8]. Computed tomography or invasive coronary angiography should be performed when acute coronary syndrome is considered [1]. CMR allows tissue characterization and scar quantification based on the 2018 Lake Louise criteria, including edema on T2‐weighted imaging and myocardial injury on late gadolinium enhancement (LGE), T1 mapping and extracellular volume [38]. The diagnostic yield of CMR is highest when performed within the first 1–2 weeks of symptom onset [6, 38]. However, CMR‐negative myocarditis may occur when imaging is performed too early (before sufficient myocyte necrosis has developed), when technical factors such as tachyarrhythmias degrade image quality or when patchy inflammation escapes spatial coverage [2]. Fluorodeoxyglucose positron emission tomography (FDG‐PET) can provide complementary information, particularly when cardiac sarcoidosis or systemic inflammatory disease is suspected [5, 39]. However, these techniques are often not feasible in clinically unstable patients due to logistic and technical constraints. In a large cohort of acute myocarditis cases, CMR was performed only in 45% of patients with fulminant myocarditis, mostly after partial recovery, within a median of 15 days from admission, compared with 4 days in non‐fulminant cases [30].

EMB remains the gold standard and is recommended in patients with fulminant myocarditis, advanced cardiac conduction disturbances or ventricular arrhythmias or failure to respond to medical therapy within 1–2 weeks [19, 40]. EMB allows determination of subtypes, including lymphocytic, eosinophilic and giant‐cell myocarditis, each carrying distinct prognostic and therapeutic implications [3, 4]. The highest diagnostic yield is within 2 weeks of symptom onset [41]; however, patchy myocardial involvement may limit sensitivity. The Dallas criteria define myocarditis based on inflammatory cell infiltration, nonischemic myocyte death and immune cells near necrotic myocytes [42]. Immunohistochemistry‐based criteria complement the Dallas criteria, defining myocarditis as ≥ 14 leukocytes/mm^2^ (including up to 4 monocytes/mm^2^) with ≥ 7 CD3‐positive T lymphocytes/mm^2^ [27]. Molecular analysis of EMB samples, including PCR‐based detection of viral genomes, can complement histology by identifying potential infectious triggers. However, these techniques may not be readily available in all centers, and the results require cautious interpretation, as viral DNA/RNA, particularly parvovirus B19 or HHV‐6, may reflect latent or incidental persistence rather than causal infection [6]. The presence of viral genome in the absence of inflammatory infiltrates is not diagnostic of myocarditis, and the results may not affect the treatment strategy and/or the response to anti‐inflammatory therapies [2]. EMB can be performed under fluoroscopic guidance from the right ventricle [43], by electroanatomic voltage mapping [44] or through echo‐guided puncture of the jugular vein [45]. In experienced centers, EMB presents low rates of complications (supraventricular arrhythmias, pericardial effusion or tamponade due to perforation and transient heart block) [41, 46] both in adults and in children [47]. The Seaport criteria have been proposed as an updated framework for lymphocytic myocarditis with a graded classification and the ‘SITUS’ category, though outcome validation is still lacking [48]. Genetic predisposition is now firmly established to explain a subset of myocarditis cases [49]. Genetic testing could be prioritized for those with recurrent myocarditis or other risk factors for inherited cardiomyopathy.

Beyond conventional cardiac biomarkers, novel circulating markers are under investigation for the diagnosis of acute myocarditis. Circulating microRNAs, particularly hsa‐miR‐Chr8:96 and miR‐721, have shown high diagnostic accuracy in distinguishing acute myocarditis from acute myocardial infarction and healthy controls [50]. Soluble ST2 (sST2), a marker of myocardial fibrosis and inflammation, has emerged as a promising tool for identifying fulminant presentations. These biomarkers may complement troponin and CMR, though further validation in larger prospective cohorts is warranted [51].

## 5 Management and Treatment

### 5.1 Pharmacological Therapy

Medical therapy is based on the severity of presentation. According to the European Society of Cardiology (ESC) [5] and the ACC recommendations [2], the main goal of treatment in acute myocarditis should be the management of arrhythmias and heart failure and, where an underlying etiological process/condition is suspected, a cause‐targeted therapy.

Although most patients with acute myocarditis spontaneously recover, patients with hemodynamically stable heart failure should be managed according to general heart failure guidelines [52]. The role of non‐steroidal anti‐inflammatory drugs (NSAIDs) varies by clinical phenotype. In myopericarditis with predominant pericardial involvement, NSAIDs combined with colchicine are first‐line therapy for symptom relief. In isolated myocarditis, their role remains uncertain, while they are generally not recommended in complicated myocarditis, due to the potential risk of exacerbating heart failure [2].

Despite the debate on whether using immunosuppressants when the virus genome is detected at the heart level, an increasing amount of data questions the direct involvement of viruses in the development of cardiac inflammation, considering the common occurrence of these viruses in individuals who do not have myocarditis and the frequent detection in EMB samples of the viral genome of previous latent infections [53]. Furthermore, targeting the viral infection in cases of acute viral myocarditis (mainly through immunomodulation with Interferon‐β therapy) has not yet been shown to be effective in randomized clinical trials (RCTs) [54].

Regardless of the initial trigger, an abnormal immune‐mediated response that causes tissue damage and chronic inflammation is considered central. In this perspective, although not clearly supported by evidence from RCTs, the American Heart Association (AHA) [19] proposes, before further diagnostic testing (including EMB) and in case of high suspicion of immune‐mediated myocarditis, early use of high‐dose intravenous corticosteroids to reduce the risk of progression to fulminant disease with haemodynamic compromise.

In fact, after the lack of conclusive evidence showing benefits of corticosteroid treatment in acute myocarditis [55], current recommendations suggest consideration of a corticosteroid‐based regimen as first‐line treatment of complicated or fulminant acute myocarditis associated with systemic autoimmune disorders or specific histology (i.e., cardiac sarcoidosis, eosinophilic, lymphocytic, giant cell myocarditis or ICI‐myocarditis) [1]. When immunosuppression is indicated, particularly in virus‐negative acute myocarditis, treatment is typically driven by both aetiology and clinical presentation and most often consists of high‐dose corticosteroids plus a steroid‐sparing agent (e.g., azathioprine, cyclosporine, mycophenolate mofetil or tacrolimus). In giant cell myocarditis, treatment requires aggressive immunosuppression, with cohort studies suggesting that early combination immunosuppression is associated with improved transplant‐free survival [56, 57]. Therefore, immunosuppression is recommended for specific histological subtypes (eosinophilic, giant cell myocarditis, cardiac sarcoidosis) and autoimmune myocarditis, based on observational cohort data and expert consensus; randomized trial evidence remains limited and standardized protocols are lacking [2, 3, 5, 58, 59].

ICI–associated acute myocarditis [60] is commonly treated by high‐dose corticosteroids and withdrawal of ICI therapy. Additionally, abatacept, a CTLA‐4 (cytotoxic T‐lymphocyte antigen‐4) agonist, has been increasingly reported as a rescue therapy in severe cases [61], and RCTs are currently evaluating the efficacy and safety of abatacept in ICI‐myocarditis (NCT05335928) and in rheumatoid arthritis‐associated myocarditis (NCT03619876).

Anti‐IL‐5 agents, such as mepolizumab and benralizumab, are increasingly used in selected immune‐mediated conditions such as eosinophilic granulomatosis with polyangiitis (EGPA) or hypereosinophilic syndromes [62, 63]. At the same time, secukinumab (IL‐17A‐neutralizing antibody) has been recently used with efficacy in patients with psoriasis and IL‐17‐related myocarditis [22].

Given recent evidence suggesting a central role of interleukin‐1 (IL‐1) in the pathogenesis of the inflammatory process and electrical instability associated with acute myocarditis [64], and the anecdotal reports of beneficial effects of IL‐1 blockade with anakinra [65, 66, 67], the ARAMIS (Anakinra vs. Placebo for the Treatment of Acute Myocarditis) trial [68] was designed to investigate the superiority of the IL‐Ra anakinra in addition to standard of care in acute myocarditis. Results from the ARAMIS trial confirmed the safety profile of anakinra in acute myocarditis, without clear evidence on its capacity to reduce the complications of acute myocarditis [69]. However, this may be explained by the selection of a low‐risk cohort and the short duration of therapy (median duration of 2 days) in the studied population. Further studies are needed to clarify the role of IL‐1 blockade in myocarditis, particularly in light of signals of benefit in small case series [66, 67, 70, 71]. A prospective cohort treated with anakinra for a median of 11 months showed a significant reduction in major adverse events during active IL‐1 blockade versus off‐treatment phases, with 7 of 8 deaths occurring outside the treatment window [72]. The ARCHER trial, testing the effect of a pharmaceutically produced cannabidiol, did not meet the primary endpoints of change in left ventricular ejection fraction; however, cannabidiol was well tolerated and was associated with reductions in LV mass [73]. Overall, these targeted anti‐inflammatory strategies are supported mainly by mechanistic rationale, disease‐specific experience, case reports or small case series and should generally be considered investigational or appropriate only for selected subtypes, severe or refractory presentations and specialized centers with multidisciplinary management.

Certain conditions can present as acute myocarditis or mimic it but have distinct etiologies, prognoses and treatment implications, and their early recognition often changes therapeutic strategy. In particular, myocarditis occurring in the setting of systemic immune‐mediated inflammatory diseases (e.g., systemic lupus erythematosus, systemic sclerosis, vasculitis or inflammatory bowel disease) benefits from early rheumatologic co‐management [74]. First‐line therapy typically includes corticosteroids to control myocardial and systemic inflammation, while steroid‐sparing immunosuppressants such as mycophenolate mofetil, azathioprine, methotrexate or rituximab can be considered according to the underlying systemic disease and the severity of cardiac involvement [74, 75]. Conversely, recurrent myocarditis‐like episodes should prompt evaluation for an underlying genetic cardiomyopathy, particularly DSP‐related disease, which is characterized by a high risk of heart failure progression and life‐threatening ventricular arrhythmias [76, 77]. Management includes guideline‐directed medical therapy, genetic counseling for patients and relatives and consideration of ICD implantation [78]. Anti‐inflammatory strategies, including corticosteroids, mycophenolate mofetil or IL‐1 inhibition, may have a role in selected cases [79].

**FIGURE 3:**
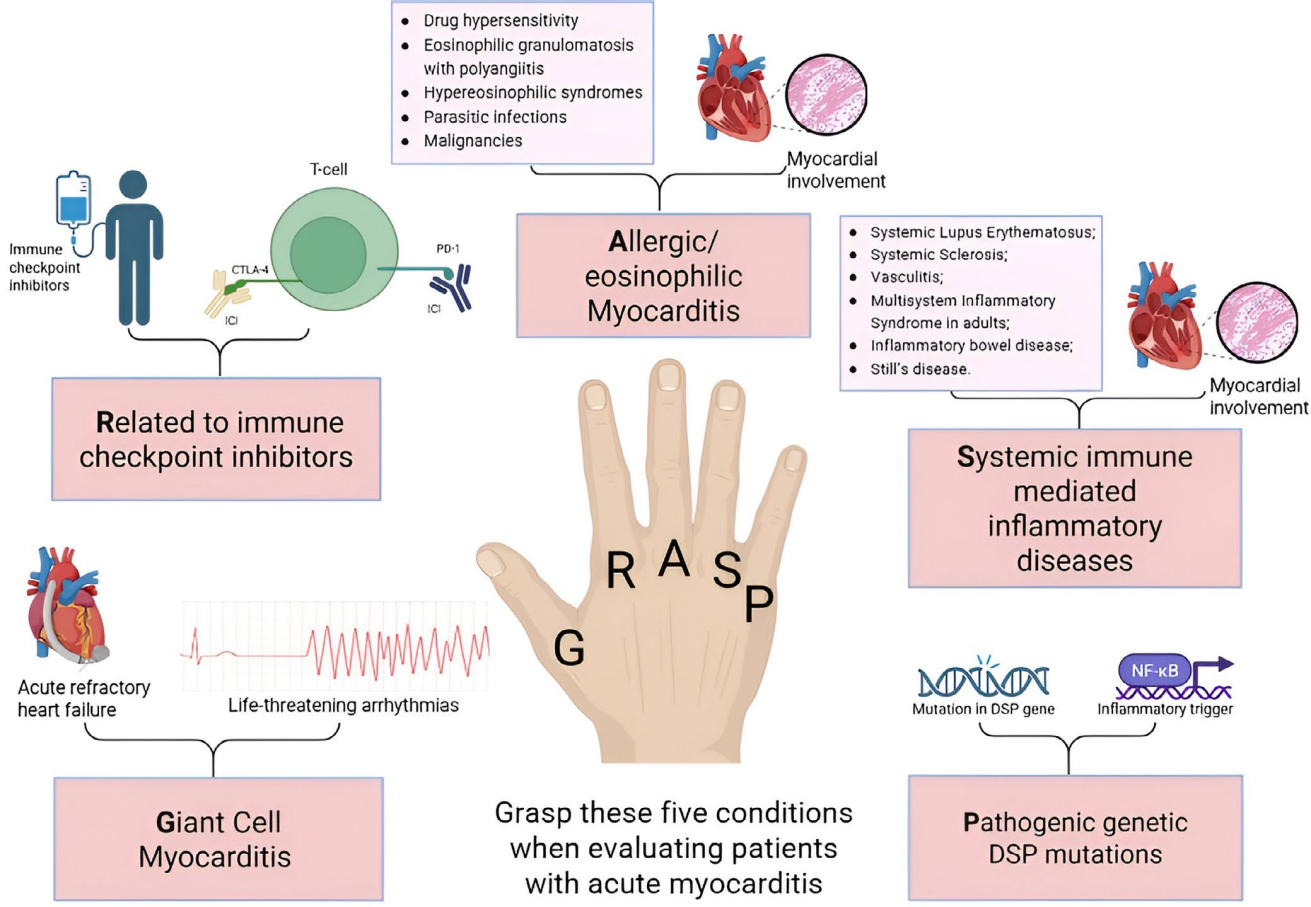
GRASP mnemonic for complex myocarditis presentations and look‐alikes. The GRASP mnemonic is presented as a practical clinical memory aid to support the early recognition of complex or atypical presentations of myocarditis and important mimickers requiring distinct diagnostic and therapeutic approaches. G (Giant cell myocarditis) is characterized by rapidly progressive heart failure and life‐threatening arrhythmias, often requiring urgent diagnosis and aggressive management. R (Related to immune checkpoint inhibitors) highlights myocarditis associated with cancer immunotherapy, mediated by T‐cell activation and immune dysregulation. A (Allergic/eosinophilic myocarditis) includes hypersensitivity reactions, eosinophilic granulomatosis with polyangiitis, hypereosinophilic syndromes, parasitic infections and malignancy‐related eosinophilia. S (Systemic immune‐mediated inflammatory diseases) encompasses connective tissue and inflammatory disorders such as systemic lupus erythematosus, systemic sclerosis, vasculitis, adult‐onset Still's disease and inflammatory bowel disease, which may involve the myocardium. P (Pathogenic genetic mutations) refers to inherited cardiomyopathies, particularly desmosomal protein (e.g., DSP) mutations, that can present with inflammatory phenotypes and mimic myocarditis. Recognition of these entities is essential, as they often require targeted investigations (including endomyocardial biopsy and advanced imaging) and tailored treatment strategies beyond standard supportive care. CTLA‐4, Cytotoxic T‐lymphocyte‐associated antigen 4; DSP, Desmoplakin; ICI, Immune checkpoint inhibitor; NF‐κB, Nuclear factor kappa B; PD‐1, Programmed cell death protein 1 (Created with BioRender.com). Readapted from Marchetta et al. [75].

To support timely recognition of these higher‐risk scenarios, the GRASP mnemonic has been recently proposed: Giant cell, Related to immune checkpoint inhibitors, Allergic/eosinophilic, Systemic immune‐mediated inflammatory disease, Pathogenic genetic desmoplakin mutations (Figure 3) [75].

### 5.2 Non‐Pharmacological Interventions

#### 5.2.1 Mechanical Circulatory Support and Heart Transplantation

While most patients with myocarditis have an uncomplicated course, about 2%–9% have hemodynamic instability requiring inotropic support or mechanical circulatory support (MCS) [1]. MCS and left ventricular assist device (LVAD) can be used as a bridge to cardiac recovery or to heart transplant in patients with complicated or fulminant myocarditis [80, 81, 82].

In a retrospective study in patients with fulminant myocarditis, patients treated with veno‐arterial extracorporeal membrane oxygenation (VA‐ECMO) had a poor prognosis, with freedom from death, durable LVAD and heart transplant of 66% at 90 days [83].

Data from the National Inpatient Sample between 2016 and 2019 identified 38,300 hospitalizations for myocarditis, of which 3490 (9.1%) had cardiogenic shock [84]. The most common form of MCS was extracorporeal membrane oxygenation. In another registry, among 850 patients with suspected acute myocarditis supported with peripheral VA‐ECMO, in‐hospital mortality was lower than in VA‐ECMO all‐comers, with nearly two‐thirds surviving to discharge [82]. Despite the growing use of temporary MCS in fulminant myocarditis, evidence remains limited regarding optimal device selection and timing. VA‐ECMO is frequently employed in centers with experience, given rapid deployment and effective biventricular support, yet short‐term outcomes remain suboptimal. Adjunctive LV unloading with axial‐flow devices (e.g., Impella) may reduce LV distension and potentially facilitate myocardial recovery [85]. Analyses of adult transplant candidates in the United Network for Organ Sharing database showed patients with myocarditis listed for transplant were younger, more critically ill, but with overall comparable survival with patients without myocarditis [86]. Post‐transplant rejection, re‐transplantation and survival rates were similar in patients with myocarditis compared to other cardiomyopathies.

#### 5.2.2 Implantable and Wearable Cardioverter Defibrillators

Rates of ventricular arrhythmia recurrence and mortality are high in patients with sustained ventricular arrhythmias during acute myocarditis. Although ICD implantation has traditionally been deferred to allow for myocardial recovery, the 2022 ESC Guidelines recommend ICD implantation prior to hospital discharge for patients with hemodynamically unstable sustained VT or ventricular fibrillation during the acute phase [87]. While ICDs have historically been recommended only in the chronic phase of myocarditis, following the development of cardiomyopathy or as secondary prevention after a sustained ventricular arrhythmia, recent studies have found that ventricular arrhythmias are common during the acute phase as well [88].

In a retrospective analysis of 69 patients with acute myocarditis and documented ventricular arrhythmias during acute illness, 27 patients (39%) had sustained ventricular arrhythmia or death during a 5.5‐year follow‐up [89], with initial monomorphic VT and predischarge LV dysfunction independently associated with worse outcomes. Similarly, another retrospective analysis revealed that 37% of patients with acute myocarditis (AM) had major arrhythmic events after discharge at a median of 8 months, with 60% occurring within the first year [90].

Since myocarditis is a potentially reversible condition, a wearable cardioverter‐defibrillator may be considered as a bridging strategy in patients with a potentially transiently increased arrhythmic risk. In a multicenter registry of 124 myocarditis patients, the device documented nonsustained VT in 9.7% and sustained VT in 6.5%, with appropriate shocks in 2.4% and a low inappropriate shock rate (0.8%) [91].

Genetic variants that predispose to myocarditis can carry important clinical implications. In patients with desmoplakin‐related cardiomyopathy, the presence of left ventricular ejection fraction < 45% and LGE on CMR may justify ICD implantation for primary prevention of SCD^78^.

### 5.3 Recovery, Follow‐Up and Patient Counseling

Current guidelines recommend abstinence from competitive sports and strenuous physical activity for 3–6 months, with return to activity contingent on symptom resolution, normalization of LVEF, resolution of inflammation on CMR and absence of clinically relevant arrhythmias [2, 5]. However, the 2025 AHA/ACC Scientific Statement on competitive sports participation acknowledges that in athletes with preserved LV function and complete resolution of myocardial inflammation on CMR, an earlier return to activity (as early as 4–6 weeks) may be considered on an individualized basis, although this recommendation is based solely on expert opinion in the absence of prospective data [92]. Follow‐up imaging should be risk‐stratified: echocardiography at 2–4 weeks post‐discharge for all patients, repeat echocardiography at 6 months for low‐risk stage C patients and multiparametric CMR at 6 months (or 3 months in athletes) for stage D or medium‐/high‐risk stage C patients [2]. Patients should be counseled to seek urgent re‐evaluation for recurrent chest pain, dyspnea, palpitations, syncope or exercise intolerance. Alcohol and recreational drug use should be avoided, and NSAIDs are generally not recommended in complicated myocarditis due to the potential risk of exacerbating heart failure [1].

## 6 Prognosis and Complications

### 6.1 Recovery Rates and Long‐Term Outcomes

The diagnosis of myocarditis carries an overall increased risk of mortality, life‐threatening ventricular arrhythmias, heart failure and disease recurrence [2, 29]. In a registry of biopsy‐proven acute myocarditis, half of cases resolved within the first 2–4 weeks, whereas persistent cardiac dysfunction or progression to end‐stage heart failure may occur in a clinically relevant subset [2]. Fulminant myocarditis with cardiogenic shock has an estimated mortality or heart transplant at 60 days and 7 years after hospitalization of 28% and 48%, respectively [1]. In uncomplicated presentations, recovery can be rapid, with in‐hospital mortality rates of approximately 0% [8]. In a registry of 187 patients with acute myocarditis, the rate of in‐hospital death or transplantation was 25.5% in the fulminant myocarditis group vs. 0% in those with less severe presentations and after a median follow‐up of 22 months, 29% of patients with fulminant myocarditis still had an LVEF < 55% [30].

Long‐term follow‐up data reinforce this dichotomy between uncomplicated and complicated presentations, with the latter carrying a ~12% risk of in‐hospital death or heart transplantation and ~15% at 5 years. Myocarditis recurrence or ventricular arrhythmias can occur in approximately 3%–9% over a follow‐up period of 19–90 months [1].

### 6.2 Predictors of Adverse Outcomes

Key risk factors for adverse prognosis include clinical severity at presentation, i.e., symptomatic heart failure, cardiogenic shock and electrical instability (recurrent ventricular arrhythmias or advanced atrio‐ventricular block) [1, 2]. Biventricular dysfunction consistently emerges as the strongest independent predictor of death or heart transplantation. The presence of LGE, particularly when involving the anteroseptal region, has been associated with poor outcomes, whereas diffuse LGE was not an independent prognostic marker in two recent studies [29, 93]. Prognosis is also strongly influenced by aetiology and histological subtype. Giant cell myocarditis is characterized by an aggressive clinical course and risk of relapse, whereas ICI‐associated myocarditis is linked to poor outcomes due to the underlying cancer and the long‐term cardiovascular risk [94]. Patients with desmosomal gene variants (especially DSP) had a 62.3% risk of death, ventricular arrhythmias and recurrent myocarditis at 5 years compared with 17.5% in those without these gene variants [76].

### 6.3 Potential Complications

Myocarditis is a major cause of SCD and DCM in young adults. Life‐threatening complications include sustained ventricular arrhythmias, advanced atrioventricular block, cardiogenic shock and progression to chronic inflammatory cardiomyopathy [5]. Among patients presenting with life‐threatening ventricular arrhythmias, characteristics associated with SCD included sustained ventricular tachycardia, fibrosis involving ≥ 2 myocardial segments and absence of edema on initial CMR [90]. Prolonged and severe inflammation can lead to extensive and irreversible fibrosis with symptoms of advanced heart failure or refractory arrhythmias, with patients requiring mechanical circulatory or inotropic support at the highest risk. In addition, selected etiologic subtypes, notably eosinophilic myocarditis, can be associated with a prothrombotic state and intracardiac thrombosis [75, 95].

## 7 Ongoing Trials and New Therapies

The treatment landscape for myocarditis is evolving, with no universally accepted guidelines due to the complex mechanisms involved (Table 1). The MYTHS (MYocarditis THerapy With Steroids) trial (NCT05150704) aims to evaluate the efficacy of intravenous corticosteroid therapy in patients with acute myocarditis complicated by acute systolic heart failure or cardiogenic shock [96]. The IMPROVE‐MC trial is designed to evaluate the efficacy and safety of a 12‐month treatment with prednisone and azathioprine in patients with biopsy‐proven virus‐negative myocarditis or inflammatory cardiomyopathy, reduced left ventricular ejection fraction (LVEF ≤ 45%) and ≥ 3‐months history of symptoms [97]. CMP‐MYTHiC is a single‐blind randomized trial evaluating whether colchicine reduces myocardial inflammation in noninvasively diagnosed inflammatory cardiomyopathy following acute myocarditis [98]. Two ongoing trials are evaluating abatacept for ICI–associated myocarditis. ACHLYS (NCT05195645) is a phase 2 study designed to identify an abatacept regimen that achieves rapid and sustained CD86 receptor occupancy in patients with severe ICI‐myocarditis. ATRIUM (NCT05335928) is a phase 3, randomized, double‐blind, placebo‐controlled trial testing whether abatacept, compared with placebo, reduces major adverse cardiac events in hospitalized patients with ICI‐associated myocarditis. The CHASM‐CS‐RCT (Cardiac Sarcoidosis Multi‐Center Randomized Controlled Trial) is designed to evaluate the efficacy and safety of a low‐dose prednisone and methotrexate combination therapy compared to standard‐dose prednisone in treating active cardiac sarcoidosis [99]. Additionally, novel therapies targeting specific immune pathways, such as TH_17_ cell research, are under investigation, with potential implications for future myocarditis management (Table 2). These distinct trials underscore the need for a stronger emphasis on clinical trial enrollment and for coordinated efforts to standardize immunosuppressive strategies, particularly in fulminant myocarditis, where practice remains heterogeneous and evidence is limited.

**TABLE 1:** Evidence framework for selected therapies in acute myocarditis.

Therapy	Indication	Level of evidence	Key findings
NSAIDs/Aspirin	Myopericarditis with preserved LVEF	Observational data (case–control)	Safe in patients with normal LVEF. Avoid in symptomatic HF or shock
Colchicine	Myopericarditis with chest pain	Expert consensus (extrapolated from pericarditis RCTs)	Recommended with NSAIDs for chest pain and preserved LVEF; being tested in chronic inflammatory cardiomyopathy (CMP‐MYTHiC trial)
Beta‐blockers	Acute myocarditis with reduced LVEF	Observational data	Associated with freedom from cardiac death or heart transplant in one longitudinal study; caution in hemodynamic instability
GDMT for HF (ACEi/ARB/ARNI, MRA, SGLT2i)	Myocarditis with HFrEF and stable hemodynamics	Guideline‐recommended (extrapolated from HF trials)	No myocarditis‐specific RCTs; recommended per standard HF guidelines
Corticosteroids	Lymphocytic myocarditis	RCT	Myocarditis Treatment Trial (*n* = 111): no benefit over placebo in LVEF or survival. Commonly used empirically in fulminant disease despite lack of evidence
High‐dose IV methylprednisolone	Fulminant/complicated myocarditis (HF, arrhythmias, shock)	Expert consensus (AHA Scientific Statement)	Empirical use recommended by AHA when immune‐mediated myocarditis is strongly suspected, before EMB. MYTHS trial ongoing
Immunosuppression (tailored, multi‐agent)	Giant cell myocarditis	Cohort studies (no RCTs)	Early combination immunosuppression associated with improved transplant‐free survival in retrospective cohorts
ICI withdrawal + high‐dose corticosteroids	ICI‐associated myocarditis	Case series, expert consensus	Standard first‐line approach; abatacept as rescue therapy in refractory cases. ATRIUM and ACHLYS trials ongoing
Corticosteroids ± steroid‐sparing agents	Eosinophilic myocarditis	Case series	Corticosteroids associated with lower in‐hospital mortality vs. no immunosuppression.
Anakinra (IL‐1Ra)	Acute myocarditis and inflammatory cardiomyopathy	RCT (ARAMIS) + prospective cohort data	ARAMIS: safe but no benefit in low‐risk cohort (median treatment 2 days). Prospective cohort (*n* = 42, median follow‐up 51 months): marked reduction in MAE during active IL‐1 blockade vs. off‐treatment phases
Cannabidiol	Acute myocarditis	RCT (ARCHER)	Did not meet primary endpoint (LVEF change); well tolerated; associated with reductions in LV mass
Abatacept (CTLA‐4 agonist)	ICI‐associated myocarditis	Ongoing RCTs (ATRIUM, ACHLYS)	Rescue therapy in case reports; phase 2 (ACHLYS) and phase 3 (ATRIUM) trials ongoing
Anti‐IL‐5 (mepolizumab, benralizumab)	Eosinophilic myocarditis in EGPA/HES	Case series; 1 RCT in EGPA (not myocarditis‐specific)	Used in selected cases of eosinophilic myocarditis associated with EGPA or hypereosinophilic syndromes
Secukinumab (anti‐IL‐17A)	IL‐17A‐related myocarditis (psoriasis)	Case series	Cardiac recovery reported in patients with psoriasis and IL‐17A‐correlated myocarditis

Abbreviations: ACEi, angiotensin‐converting enzyme inhibitor; AHA, American Heart Association; ARB, angiotensin receptor blocker; ARNI, angiotensin receptor‐neprilysin inhibitor; CTLA‐4, cytotoxic T‐lymphocyte‐associated protein 4; EGPA, eosinophilic granulomatosis with polyangiitis; EMB, endomyocardial biopsy; GDMT, guideline‐directed medical therapy; HES, hypereosinophilic syndrome; HF, heart failure; HFrEF, heart failure with reduced ejection fraction; ICI, immune checkpoint inhibitor; IL, interleukin; IL‐1Ra, interleukin‐1 receptor antagonist; IV, intravenous; LVEF, left ventricular ejection fraction; MRA, mineralocorticoid receptor antagonist; NSAIDs, nonsteroidal anti‐inflammatory drugs; RCT, randomized controlled trial; SGLT2i, sodium‐glucose cotransporter‐2 inhibitor.

**TABLE 2:** Ongoing clinical trials.

First author/name of the study	Starting year	Type of study/design	Target number of patients	EMB	Imaging	LVEF (%)	Intervention	Follow‐up (months)	Trial ID	Status
Birnie et al./CHASM‐CS‐RCT trial	2019	Multicentre. Randomized. Double‐blind.	194	−	+	−	Prednisone 0.5 mg kg/day for 6‐months (MAX dose 30 mg per day) or Methotrexate 15–20 mg PO, SC or IM once a week for 6‐months + Folic Acid 2 mg OD for 6 months + Prednisone 20 mg day for 1 month, then 10 mg OD for 1 month, then 5 mg OD for 1 month, then STOP	6	NCT03593759	Recruiting
Ammirati et al./MYTHS trial	2021	Multicentre. Randomized. Single‐blind. Pragmatic design.	288	−	−	< 41	High‐dose methylprednisolone vs. placebo for 3 days.	6	NCT05150704	Recruiting
Ozierański et al./IMPROVE‐MC trial	2022	Multicentre. Randomized. Double‐blind.	100	+	+	≤ 45	Prednisone and AZA vs. placebo for 12 months.	12	NCT04654988	Recruiting
Ederhy et al./ACHLYS trial	2022	Single centre. Randomized. Double‐blind.	20	−	+/−	< 50	Abatacept (CTLA‐4 fusion protein) at 3 different IV regimens (10 mg/kg vs. 20 mg/kg vs. 25 mg/kg every week) for 3 weeks.	12	NCT05195645	Completed
Reynolds et al./ATRIUM	2022	Multicenter Randomized Double‐blind	390	+/−	+	−	Abatacept IV over 30 min at baseline, 18–24 h later and at 14 and 28 days later vs. placebo	6	NCT05335928	Recruiting
Ammirati et al./CMP‐MYTHiC	2023	Multicenter Single‐blind Randomized	80	−	+	< 50	Colchicine 1 mg daily (or 0.5 mg daily if weight < 70 kg) from randomization for 180 days (6 months) vs. placebo	6	NCT06158698	Recruiting

Abbreviations: AZA, azathioprine; CTLA‐4, cytotoxic T‐lymphocyte associated protein 4; EMB, endomyocardial biopsy; IL, interleukin; IV, Intravenous; LVEF, left ventricular ejection fraction; MMF, mycophenolate mofetil; NS, not specified.

## 8 Conclusion

Acute myocarditis remains a heterogeneous syndrome in which prognosis and treatment are driven by clinical severity, aetiology and immune phenotype. A practical approach should combine early risk stratification with selective use of advanced imaging, EMB and aetiology‐directed therapy. Future studies should define which patients benefit from immunomodulation and identify biomarkers that reliably guide prognosis, follow‐up and therapeutic selection.

## Author Contributions

M.G. conceived the review, performed the literature search, drafted the manuscript and coordinated the contributions of all authors. M.G.D.B., M.M., N.P., A.B., A.V., D.M., G.E. and J.K. contributed to the literature search and drafting of specific sections. A.A. supervised the project, contributed to the draft, provided critical revision of the manuscript for important intellectual content and gave final approval of the version to be submitted. All authors reviewed and approved the final manuscript.

## Funding

The authors have nothing to report.

## Ethics Statement

This article is a clinical review based on previously published literature and does not involve any new studies of human participants or animals performed by the authors. Therefore, approval by an ethics committee/institutional review board and informed consent were not required.

## Conflicts of Interest

Dr. Abbate has served as a consultant for Kiniksa, Monte Rosa and Novo Nordisk. The other authors have no other disclosures.

## Data Availability

Data sharing not applicable to this article as no datasets were generated or analyzed during the current study.
